# Legionnaires’ disease from a cooling tower in a community outbreak in Lidköping, Sweden- epidemiological, environmental and microbiological investigation supported by meteorological modelling

**DOI:** 10.1186/1471-2334-12-313

**Published:** 2012-11-21

**Authors:** Peter Ulleryd, Anna Hugosson, Görel Allestam, Sverker Bernander, Berndt EB Claesson, Ingrid Eilertz, Anne-Christine Hagaeus, Martin Hjorth, Agneta Johansson, Birgitta de Jong, Anna Lindqvist, Peter Nolskog, Nils Svensson

**Affiliations:** 1 Department of Communicable Disease Control and Prevention, Region Västra Götaland SE-501 82, Borås, Sweden; 2 Institute of Biomedicine, Sahlgrenska Academy, University of Gothenburg, Gothenburg, Sweden; 3 Department of Medicine, Lidköping Hospital, Lidköping, Sweden; 4 Department of Diagnostics and Vaccinology, Swedish Institute for Communicable Disease Control, Solna, Sweden; 5 Section of Clinical Microbiology, Department of Medical Sciences, Uppsala University Hospital, Uppsala, Sweden; 6 Department of Clinical Microbiology, Skövde Hospital, Skövde, Sweden; 7 Department of Veterinary, County Administrative Board, Västra Götaland, Sweden; 8 Department of Environmental Health Office, Community of Lidköping, Lidköping, Sweden; 9 Department of Surveillance and Response Support, European Centre for Disease Prevention and Control, Stockholm, Sweden; 10 Department of Infectious Disease, Skövde Hospital, Skövde, Sweden; 11 At the time the study was conducted: Department of Epidemiology, Swedish Institute for Infectious Disease Control, Solna, Sweden

**Keywords:** Legionnaires’ disease, Outbreak, Epidemiology, Cooling tower, Meteorological computer models, Sequence based typing

## Abstract

**Background:**

An outbreak of Legionnaires’ Disease took place in the Swedish town Lidköping on Lake Vänern in August 2004 and the number of pneumonia cases at the local hospital increased markedly. As soon as the first patients were diagnosed, health care providers were informed and an outbreak investigation was launched.

**Methods:**

Classical epidemiological investigation, diagnostic tests, environmental analyses, epidemiological typing and meteorological methods.

**Results:**

Thirty-two cases were found. The median age was 62 years (range 36 – 88) and 22 (69%) were males. No common indoor exposure was found. *Legionella pneumophila* serogroup 1 was found at two industries, each with two cooling towers. In one cooling tower exceptionally high concentrations, 1.2 × 10^9^ cfu/L, were found. Smaller amounts were also found in the other tower of the first industry and in one tower of the second plant. Sero- and genotyping of isolated *L. pneumophila* serogroup 1 from three patients and epidemiologically suspected environmental strains supported the cooling tower with the high concentration as the source. In all, two *L. pneumophila* strains were isolated from three culture confirmed cases and both these strains were detected in the cooling tower, but one strain in another cooling tower as well. Meteorological modelling demonstrated probable spread from the most suspected cooling tower towards the town centre and the precise location of four cases that were stray visitors to Lidköping.

**Conclusions:**

Classical epidemiological, environmental and microbiological investigation of an LD outbreak can be supported by meteorological modelling methods.

The broad competence and cooperation capabilities in the investigation team from different authorities were of paramount importance in stopping this outbreak.

## Background

In 1976 at the American Legion Convention in Philadelphia, US, a severe pneumonia struck 182 of the attendees of which 29 where fatal [1]. The disease was called Legionnaires’ disease (LD) and the bacterium was named *Legionella pneumophila*.

Legionnaires’ disease has most often an incubation time of two to ten days. It is estimated that 0.5 - 5% of community acquired pneumonia is due to *Legionella* infection [2,3].

Numerous community-acquired outbreaks have been published, mostly emanating from cooling towers. Meteorological factors such as wind, humidity, and dispersion modelling were also discussed [4-11]. The only other known Swedish outbreak from a cooling tower also occurred in a hot and humid summer [4].

### Outbreak investigation

Three patients with clinically suspected *Legionella* pneumonia were reported 19 August 2004 by the clinicians at the Department of Medicine, Lidköping Hospital and the Consultants from the Department of Infectious Disease, Skövde Hospital to the Department of Communicable Disease Control and Prevention, Region Västra Götaland, a region with 1.5 million inhabitants. All three patients were living in Lidköping, a town with 38 000 inhabitants situated at river Lidan nearby the large Lake Vänern. The clinical suspicion of Legionnaires’ disease was based on non-responsiveness on initial standard betalactam treatment and further confirmed by urinary antigen test in two of the patients. Also, during the first half of August the hospital in Lidköping noticed a considerable increase of admissions of pneumonia patients. Health-care providers were informed about the possibility of an outbreak with LD, patient interviews and environmental sampling in the patients’ homes started, and localization of cooling towers and other aerosol producing installations began.

On 29 August a total of nine patients with a confirmed LD had been identified and next day an extended outbreak investigation team was formed with participation from the authors’ respective departments.

The objective of this report was to describe the outbreak from an epidemiological point of view and how the workup with identifying the source in order to stop the current outbreak was organised.

## Methods

### Case definition

A person living in or visiting Lidköping between 1 August and 13 September 2004 seeking medical attention with clinical symptoms suggestive of LD plus clinically or radiologically verified pneumonia was considered as a presumptive case. Presumptive cases were identified in Lidköping Hospital, Skövde Hospital, primary health care centers in Lidköping and the mandatory Swedish notification system of communicable diseases. Furthermore, a positive sputum culture or urinary antigen test was defined as a confirmed LD case. A serological ≥ fourfold change in *Legionella* antibody titre or a single antibody titre of at least 1/128 in this outbreak setting was also defined as a confirmed LD case. A serological rise to a titre of 1/64 was defined as a presumptive LD case. Confirmed and presumptive cases were included in the total number of *Legionella* cases.

### Microbiological investigation

Respiratory specimens were cultured on non-selective and selective Buffered Charcoal Yeast Extract agar (BCYEα and MWY). Isolated Legionella strains were tested for species and serogroups. Urinary antigen tests were performed using Binax NOW ICT for detection of *L. pneumophila* serogroup (sg) 1 antigen. Serology for detection of *Legionella* antibodies was performed simultaneously using an indirect immunofluorescent technique (IFAT) with in-house prepared antigens by Section of Clinical Microbiology, Uppsala University Hospital.

### Epidemiological investigation

Patients were interviewed according to a structured questionnaire including known risk factors for acquiring LD. The interviews were performed face to face by trained personnel with local knowledge of Lidköping. A map divided in small square fields was used to pinpoint where in the city the patients had been within 14 days before onset of symptoms. Repeated interviews were needed for patients with cerebral confusion and relatives were sometimes asked to complete the interviews.

### Environmental investigation

The temperature was measured and samples were taken of the water in the homes by the Environmental Health Office personnel and if necessary advice of action was given. Public water reservoirs were also inspected and sampled. Possible industrial sources for *Legionella*, mainly cooling towers, were investigated.

Water samples from showers were analyzed by membrane filtration technique and incubated in 36°C on MWY-agar (Oxoid) for 7–10 days according to ISO standard 11731–2. Samples from cooling towers were analyzed in 10-fold series after acid treatment and further spread on MWY-agar. A latex agglutination test with Dryspot (Oxoid) directly from the agar plate was used after two days incubation for the cooling tower samples for preliminary identification.

### Epidemiological typing

Bacterial isolates were cultured on BCYEα agar at 35°C for 3 days before testing.

Serological typing was performed using the Dresden panel of monoclonal antibodies (MAb) [12]. The genetic profile of patient isolates was compared to that of environmental isolates using sequence-based typing (SBT) using the genes ***fla*A,*pil*E,*asd*,*mip*,*momp*S,*pro*A,*neu*A**. A consensus method has been validated and a database for sequence type (ST) set up by The European Working Group for Legionella Infections (EWGLI) [13].

### Meteorological methods

Weather reports from nearby Såtenäs airport were assessed. ALARM (Advanced Local And Regional Modelling system), a model for spread from a point source using a combination of measurements and a database of simulated wind and turbulence fields was used, where the topography of the terrain is also included [14-17]. This system is implemented in Region Västra Götaland including three SODAR (sonic detection and ranging) plants and 10 mast measuring points. The main purpose of ALARM is to help the authorities predict spread of toxic substances, for example in case of a chemical accident.

### Ethical considerations

The work in this paper was part of an outbreak investigation. The regional departments of communicable disease control and prevention in Sweden have general consent to conduct outbreak investigations and contact tracing is routine. This is regulated by the Swedish Communicable Diseases Act (2004) and includes interviews, testing, epidemiological investigations, communicating results of the investigation etc. A specific approval for the work presented was therefore not needed in this outbreak setting.

The purpose of this work was to identify the cause of the outbreak and to implement interventions to prevent further cases, also preventing future outbreaks-to-be. Therefore, this report was not considered to be research and was therefore exempt from human subjects review.

## Results

### Cases and microbiological investigation

**Figure 1:**
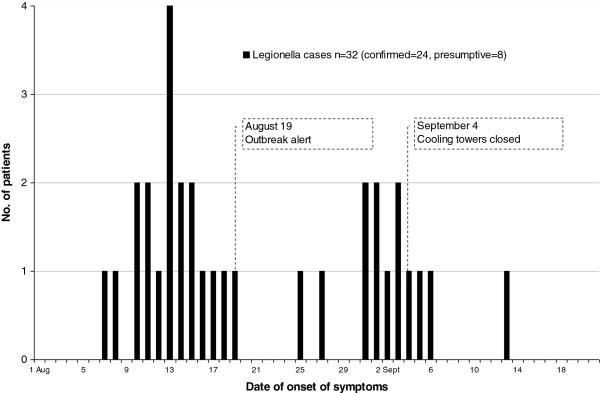
**Epidemic curve of the *Legionella *outbreak in Lidköping 2004.**

In all, 138 patients were investigated. Eighty-four were admitted to the hospital in Lidköping during the weeks of the outbreak, which was overall a more than a twofold increase in the number of hospitalized patients with pneumonia, most of them radiologically visualized, in comparison with previous years. The peak incidence of these possible cases did coincide with the two-peaked epidemic curve (Figure 1). The other patients were investigated at other hospitals or primary health care centers.

All diagnostic procedures were not performed in all patients. Sputum culture, urinary antigen test, and serology for *Legionella* were performed in 50, 72, and 97 patients respectively.

Three cases grew *L. pneumophila* sg 1. Twelve other cases showed a positive urinary antigen test for *L. pneumophila*. Of these, in all 15 patients, 14 were available for serological testing. Six cases showed a significant titre change or a single high titre ≥ 128, one was presumptive with a titre of 64 and seven were negative.

Additionally, nine patients were found to be serologically confirmed cases. Another eight patients were presumptive serologically positive and included as LD cases according to the case definition. The total number of confirmed and presumptive LD cases was 32.

The median age of the 32 cases was 62 years (range 36–88) with 22 (69%) males. Other personal risk-factors found were current tobacco smoking in 22 (69%) and severe medical illness in 12 (38%) of the patients. All patients presented with a temperature ≥ 38.0°C, 23 (72%) had symptoms of central nervous system engagement, 17 (53%) had cough and gastrointestinal symptoms, and two were submitted to assisted ventilation. Two cases died, giving a mortality rate of 6%. A detailed report on case characteristics, diagnostic yield and clinical course for the 32 confirmed and presumptive LD cases have been published separately [18].

### Epidemiological investigation

Twenty-one of the 32 cases lived in central Lidköping, seven were frequent visitors and lived in the municipal periphery, and four of the patients had only visited the municipality of Lidköping at one time within a 2–10 day period before onset of their symptoms. No common indoor exposure was found in the interviews. None of the patients confirmed any contact with whirlpools, car-washing facilities, air humidifiers/air conditioning devices at home or home-fountains.

**Figure 2:**
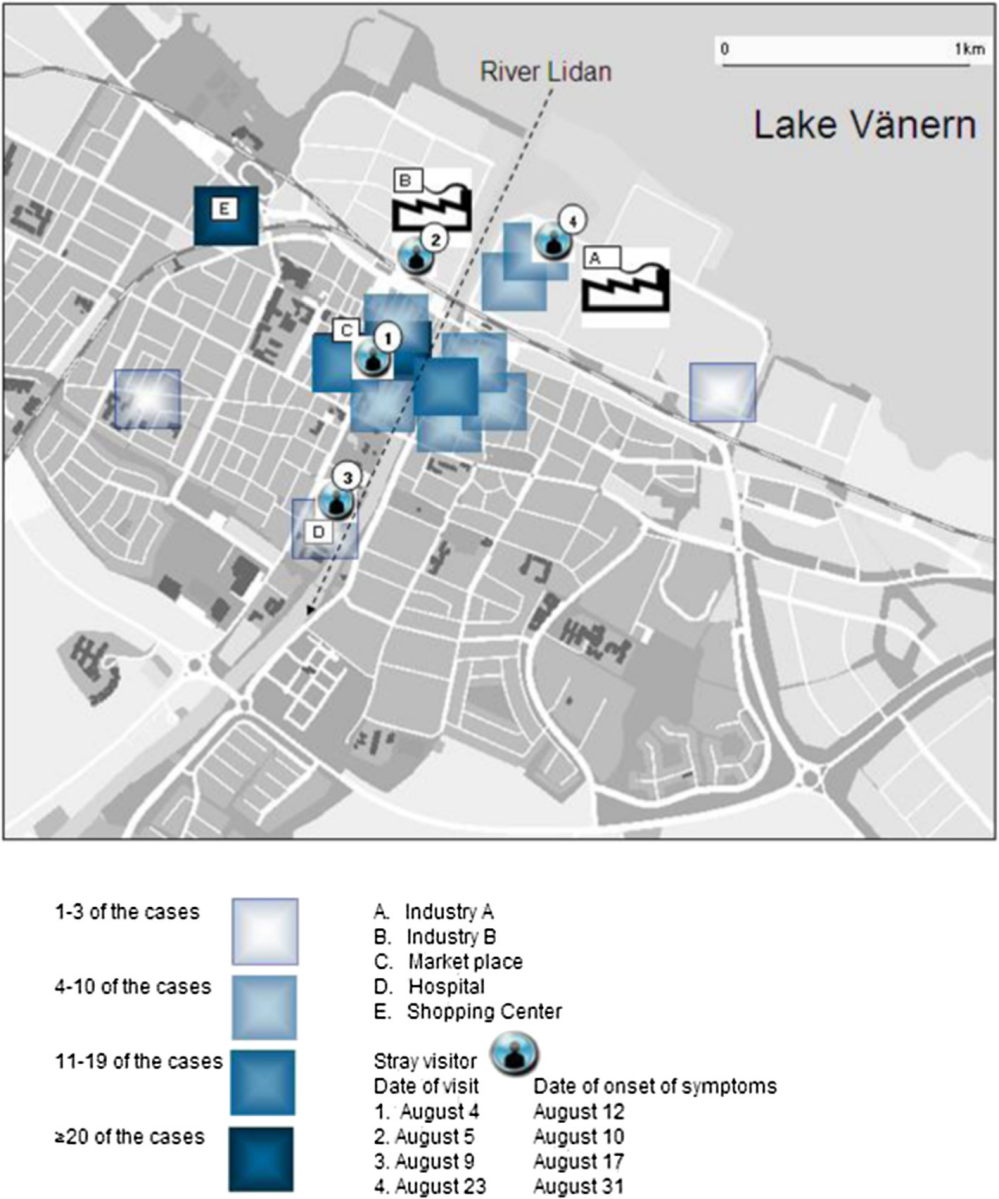
**Areas that the cases mainly had visited within two weeks before onset of symptoms in the *Legionella *outbreak in Lidköping 2004.** The Market place is the central part in town. The four stray visitors points of visit are indicated.

Figure 2 shows the areas that the residents mainly had visited in the central town and the only places where the stray visitors had stopped. The duration of the stay of the four stray visitors varied between a half and two hours. Their incubation period was 5, 8, 8 and 8 days, respectively. Ten patients had visited the supermarket with a mist machine. Cooling tower 1 and 2 at Industry A had been closed for vacation two weeks until late July.

### Environmental investigation

A total of 61 environmental samples for *Legionella* were processed. Initially, investigations of the showers grew *Legionella* in two of six patient homes with 3500 and 150 cfu/L respectively, in the samples taken after flushing. The identification showed other serotypes than found among patient isolates. In a mist machine for vegetables at the supermarket (Figure 2), a concentration of 79 000 cfu/L of *L. pneumophila* sg 1 was found. This device had been out of use for 4 days when sampled and the amount of bacteria found in the sample does not reflect the situation when it was in use.

All cooling towers in the city, fifteen towers in eight industries, were sampled for culture. Outdoor fountains and drinking water were negative.

Three cooling towers from two industries (A and B) were positive for *L. pneumophila* sg 1*.* After only 48 hours incubation of the first samples from the suspected Industry A, cooling tower 1 we spotted thousands of *Legionella* colonies and a latex agglutination test was positive for *L. pneumophila* sg 1. Further quantification showed growth of 1 200 000 000 cfu/L. The other two positive cooling towers had concentrations of *Legionella* between 100 000 – 600 000 cfu/L, all of them exceeding the upper action limit given by EWGLI at 10 000 cfu/L [3].

### Epidemiological typing

MAb subgrouping showed that two patient isolates belonged to subgroup Benidorm and one to Bellingham. Serogroups and subgroups of patient and environmental isolates are shown in Table 1.

**Table 1:** ***Legionella *outbreak isolates from patients and environment in Lidköping 2004**

**Isolate origin**	**Legionella cfu per L**	**Legionella pneumophila serogroup**	**Monoclonal subtype (Dresden panel)**	**Seven gene SBT (*fla*A, *pil*E, *asd*, *mip*, *momp*S, *pro*A, *neu*A)**	**Sequence type**
**Patient I**		**1**	**Benidorm**	**6, 10,2,28,9,4,6**	**639**
**Patient II**		**1**	**Benidorm**	**6, 10,2,28,9,4,6**	**639**
Patient III		1	Bellingham	7,6,17,3,13,11,11	59
**Cooling tower 1, Industry A**	400 000 000*	**1**	**Benidorm**	**6, 10,2,28,9,4,6**	**639**
	800 000 000*	3			
**Cooling tower 2, Industry A**	**300 000**	**1**	**Benidorm**	**6, 10,2,28,9,4,6**	**639**
		1	Benidorm	7,6,17,3,13,11,11	59
Cooling tower 3, Industry B	300 000	4	Portland		
Cooling tower 4, Industry B	600 000	1	Olda	1,4,3,1,1,1,1	1
		1	Bellingham	7,6,17,3,13,11,11	59
Mistmachine, supermarket	79 000	1	Benidorm	7,6,17,3,13,11,11	59
Shower at home Patient II	3 500	6			
Shower at home Patient III	150	12			

* Total concentration of Legionella was 1200 000 000 cfu per L in the suspected source CT1 at Industry A. Ratio of 48 sub cultivated colonies.

The two patient Benidorm (I, II) isolates (ST639) were genotypically quite different from the patient Bellingham (III) isolate (ST59). Both STs could be found in the suspected cooling towers of Industry A, which was suspected as a source of infection. However, the environmental isolate that was genotypically identical with the patient Bellingham strain reacted as a Benidorm using the MAb panel. It has been shown previously that Benidorm and Bellingham strains that have identical genetic profiles may occur in the same water system [19]. Based on the ST we regard Industry A/cooling tower 2 as the likely source of the infection in the patient with the Lp1/Bellingham/ST59 strain.

### Meteorological results

**Figure 3:**
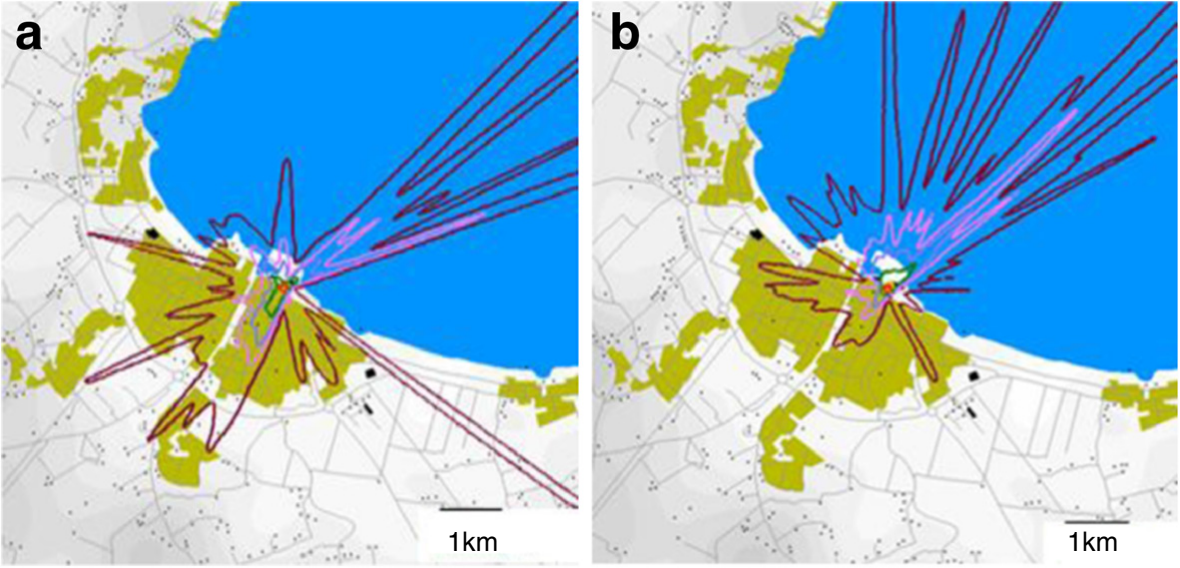
**a and 3b demonstrate the modelled main concentration plume from the suspected cooling tower as an average during the first and the last half part of the *Legionella *outbreak in Lidköping 2004, respectively.** Bright red color stands for the highest concentration.

**Figure 4:**
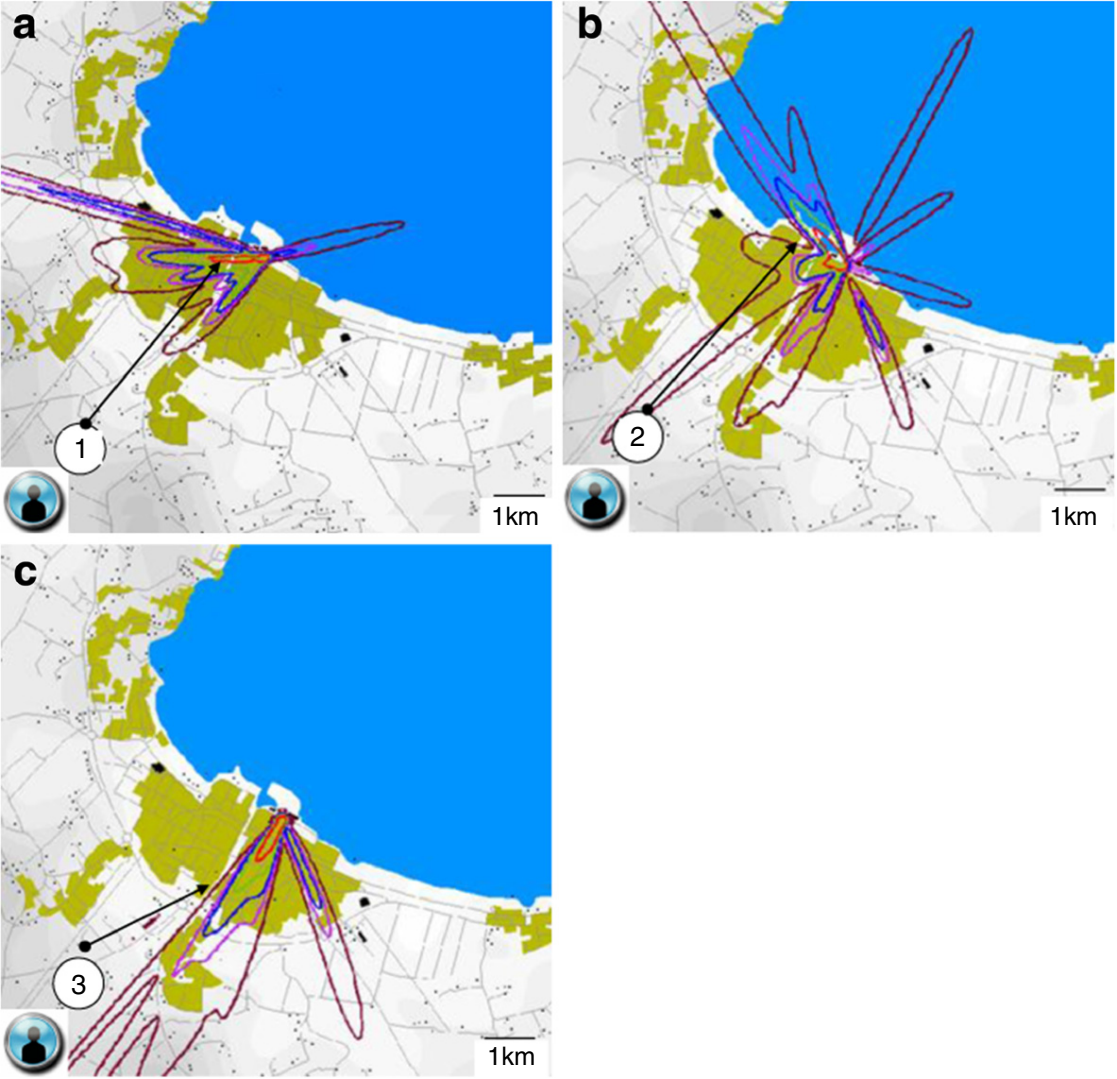
**a, 4b and 4c demonstrate the modelled main concentration plume from the suspected cooling tower during the short time three of the four stray visitors visited Lidköping during the *Legionella *outbreak 2004.** The fourth stray visitor spent two hours within 50 meters from the cooling tower. Bright red color stands for the highest concentration. Figure 4a, Stray visitor 1 August 04. Figure 4b, Stray visitor 2 August 05. Figure 4c, Stray visitor 3 August 09.

The last weeks before and during the outbreak was a hot and humid summer period according to nearby Såtenäs airport weather reports. Prevailing wind direction during the outbreak was (south-west) coast to lake (Figure 3a, b). The actual days when the four stray visitors were in Lidköping, the main wind direction was (north-east) towards the coast and town centre. The main concentration plume from the ALARM modelling was pointing towards the precise areas these four people were visiting. Figure 4a, b, c visualize the ALARM model for three of the patients. The fourth stray visitor spent two hours within 50 meters from the cooling tower 1, observing the condensed aerosol drifting in his direction (Figure 2).

### Investigation progress

The two cooling towers at Industry A were closed 3 September and decontaminated. Industry B closed their cooling towers for decontamination 4 September. These decisions were based on the finding of the huge growth in one of the cooling towers, the patient information of an intermittent unpleasant smell blowing in the wind from a power plant adjacent to Industry A, and the four stray visitors. The last onset of symptoms of a confirmed case was 13 September, within the incubation time (Figure 1).

Initially, there was a certain fear and pressure from the public and media on the authorities for some kind of action. No general closing or other action was taken until 3 September. The progress of the investigation was thoroughly reported in press releases, put together by the investigation team at telephone conferences on a regular basis, first every two days, and later more infrequent. Questions regarding the environmental investigation were answered by one person at the local Environmental Health Office and questions about the disease or the patients were answered by one specified person from the Department of Communicable Disease Control and Prevention. A lot of telephone calls from the public were also answered by respective authority and the local healthcare.

## Discussion

An industrial cooling tower was the probable source for this *Legionella* outbreak with aerosol spread that included at least 32 patients with two fatal outcomes. The cooling tower was of the open-type without a drip-eliminator where condensed steam was released with a very high concentration of *L. pneumophila* sg 1, subtype Benidorm belonging to the virulent MAb 3–1 group.

There was a concomitant increase in patients with lower respiratory tract infections, the majority of these admitted to hospital (Figure 1). It is evident in this study that not all true cases are found or detected with our standard diagnostic tests, which has also been discussed in earlier LD outbreak reports [5,6]. After recognition of the outbreak 19 August, there was an increased tendency of the public to attend healthcare and the alertness possibly rendered more patients with suspected pneumonia to be admitted to hospital. This phenomenon could at least to some extent explain the second peak. However, much of the surplus of pneumonia cases compared to the expected incidence could represent LD cases not confirmed by the available diagnostic tests. The importance of informing the public in outbreak management is pointed out in another report [5].

One limitation of this investigation is that no case–control analysis was done. Such a study was initially discussed but refrained from mainly two reasons. First, it could be difficult to find a control group in this epidemic that was spread in all Lidköping of a disease where possibly only one in 1000 exposed will contract the disease [2]. Second, the time and effort in performing a case–control analysis would possibly jeopardize a fast finding and closing of the source.

*Legionella* culture from sputum was positive in three cases out of 50 cultures sampled from 106 patients. As the first wave of onset of lower respiratory tract infection already peaked before the recognition of the outbreak (Figure 1), a lot of emphasis of the clinicians was put in obtaining these cultures. An early identification of patient isolates is crucial for the epidemiological typing comparison with available environmental isolates. But, given the low diagnostic yield, sputum culture for confirmation of LD has limited importance from a clinical point of view compared to the urinary antigen test and sputum PCR test if available.

The patient interviews were time-consuming as many of the patients experienced some degree of confusion. A very good local knowledge of the interviewers was of importance, particularly in the mapping of where the patients had been during the 14 days prior to onset of symptoms. As described in most outbreaks, the majority of the patients were elderly, males, and tobacco smokers [2,3].

Finding “outlaying cases” such as the four stray visitors in Lidköping was of great help in the epidemiological investigation, and later combined with the wind modelling, reinforced the data presented from the environmental cultures and typing.

The finding of positive *Legionella* cultures from the environment is expected. A mixture of more than one *Legionella* species or subtype in the same sample is also common [3,4]. The number of subtypes found in each sample was maybe underestimated in reports before modern genotyping methods were available [3]. Another confounding factor in typing is the difficulties in selecting enough number of colonies from the primary culture. All typing methods have their advantages and disadvantages and each method contribute in its own way to the puzzle of linking patient- and environmental isolates to each other.

Both sequence types isolated from the patients and Industry A could also be found at other locations in Lidköping, possibly due to a common spread from open water or the water system into the surroundings, as expected according to other studies [20]. Cooling tower 1 in Industry A had a remarkably high concentration of *Legionella* sg 1 ST639, making it the most probable source. The lower concentration of *Legionella* sg 1 ST59 in Industry B made it less likely as the source of the outbreak. ST639 is only found in Lidköping in the SBT database at EWGLI.

Meteorological investigation and modelling can provide important information about wind conditions as aerosol spread up to many kilometers have been reported [6-11]. Different types of dispersion modelling systems are quite widespread, mainly for scientific purposes or in case of chemical or radioactive pollution.

The probable date of infection for the index cases in our investigation coincided with a change from a chilly to a rather warm humid weather, which may have played a role in the spread of the *Legionella* bacteria from the cooling tower. Some reports have stated that it is the humidity, not the heat, which increases the risk for LD outbreaks [21]. Comparing the airport weather data did not give any clear-cut explanation of the two-spiked epidemic curve. It has been suggested that high temperatures and high humidity can influence infective nuclei in an aerosol to spread more easily [9]. Other factors such as biological material within aerosol-producing water sources and biofilm production may be of importance preceding an outbreak [3]. We did notice a lot of biofilm formation inside and on the cooling tower 1 at Industry A and also on the walls of the surrounding buildings.

Outbreak investigation is always a race against time. The outbreak investigation team has to gather as many facts as possible in a short time to be able to draw a conclusion of the most probable source or sources of the outbreak. The closing or elimination of the probable source must often be expedited without waiting for all results of sampling. All in order to avoid further cases without unnecessary disturbing normal social life and enterprise. The first 48 hours after notification of a LD outbreak have been reported to be very important [22].

## Conclusions

Classical epidemiological, environmental and microbiological investigation of an LD outbreak can be supported by meteorological modelling methods.

A steady alertness for unexpected clustering of clinical cases, prompt organization of the investigation team, and a smoothly running cooperation of all included stakeholders such as the health-care, local-regional-national authorities, the public, the media, and the private enterprise were factors that facilitated the relatively fast finding and closing of the source for this outbreak. After the outbreak, an intense activity of industries all over Sweden started regarding the maintenance and control of existing cooling towers such as already existing in other countries [23].

## Misc

Sverker Bernander Deceased

## Competing interests

The study was supported by grants from the Research Fund at Region Västra Götaland and from the Research Fund at Skaraborg Hospital.

The authors declare that they have no other competing interests.

## Authors’ contributions

GA and SB carried out the sequence based typing and SB the serological typing. The other authors participated in the acquisition of patient and environmental data. All authors participated in the design, analysis and interpretation of data, critically revising the manuscript and given final approval.

## Pre-publication history

The pre-publication history for this paper can be accessed here:

http://www.biomedcentral.com/1471-2334/12/313/prepub
